# A Term Neonatal Case With Lethal Respiratory Failure Associated With a Novel Homozygous Mutation in ABCA3 Gene

**DOI:** 10.3389/fped.2020.00138

**Published:** 2020-04-17

**Authors:** Meili Wei, Haibo Fu, Aiqin Han, Liji Ma

**Affiliations:** Department of Pediatrics, Zibo Central Hospital, Shandong, China

**Keywords:** full-term neonate, ABCA3 gene, pulmonary surfactant, interstitial lung diseases, lethal respiratory distress syndrome

## Abstract

The mutations in the *ABCA3* (ATP-binding cassette transporter subfamily A member 3) gene could result in lethal respiratory distress syndrome (RDS) in neonates and interstitial lung disease (ILD) in infants and children. Here, we describe a full-term newborn who manifested respiratory distress 20 min after birth and then gradually developed hypoxemic respiratory failure and died on 53 days of life. A homozygous missense mutation (c.746C >T) was identified in exon 8 of *ABCA3* gene in the neonate by next-generation sequencing, and the mutations were inherited from parents, respectively. This homozygous mutation is the first reported to date.

## Introduction

Neonatal respiratory distress syndrome (NRDS) is a primary or secondary deficiency of pulmonary surfactant and is the primary cause of neonatal death. Survivors may have severe interstitial lung disease (ILD). ILD was a group of clinically, radiologically, and pathologically similar heterogeneous diseases, characterized by progressive oxygenation disorder and respiratory failure. ILD is extremely rare in infants and children, the prevalence is 1 per 100,000, and about 10% of ILD are caused by mutations in genes encoding pulmonary surfactant and related metabolic pathways (1–3), including surfactant proteins (SP)-B, SP-C, SP-D, ATP-binding cassette subfamily A member 3 (ABCA3), NKX2-1, TBX4, etc. There was great heterogeneity in the severity of ILD caused by *ABCA3* mutations, ranging from the fetal neonatal respiratory distress after birth to the children's ILD (4–6).

Here, we present clinical characteristics and outcomes of ILD in a neonate that correlated to homozygous missense mutation (c.746C > T) in the *ABCA3* gene.

## Case Representation

A full-term male Chinese neonate weighting 3,900 g was born (gravida 1, para 1) at 38^6/7^ weeks of gestation via cesarean section due to breech presentation. The Apgar scores at 1, 5, and 10 min were all 10. The parents were both 33-years Han Chinese and denied the family history of similar disease or any other genetic disease.

**Figure 1:**
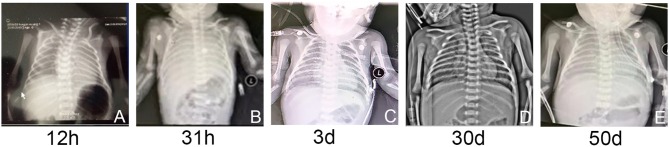
**(A–E)** The chest radiographs showed varying degrees of decreased transmittance and lung consolidation.

Respiratory distress occurred at 20 min after birth; the neonate was given humidified oxygen with face mask. Due to the progressively exacerbated respiratory distress, he was admitted to the neonatal intensive care unit. The physical examination was notable for cyanosis, grunting, tachypnea respiratory rate more than 80 breaths per minute, intercostal and subcostal retractions, reduced primitive reflexes. He was supported with nasal continuous positive airway pressure (NCPAP) with FiO_2_ of 0.25. Arterial blood gas showed a PaO_2_ of 58 mmHg. Due to the progressively deteriorated respiratory distress, FiO_2_ was gradually elevated to 0.45 at 12 h of life. Chest radiograph showed decreased transmittance in bilateral lungs (Figure 1A). At 31 h of life, he developed lethal respiratory distress with PaO_2_ of 36 mmHg. Chest radiograph showed decreased transmittance with blurred cardiac and diaphragmatic margins (Figure 1B). Echocardiography excluded structural heart disease and pulmonary hypertension. He was intubated and given high-frequency oscillatory ventilation with FiO_2_ of 0.85. After the administration of 360 mg pulmonary surfactant, his respiratory distress moderately ameliorated with FiO_2_ gradually decreased to 0.6. Blood tests indicated leukocytosis, elevations in C-reactive protein concentration (23 mg/L), procalcitonin concentration (11 ng/ml), and interleukin-6 concentration (228 pg/ml). All the results of culture including blood, urine, and cerebrospinal fluid were negative. Intravenous meropenem and immunoglobulin (1 g/kg/day) were empirically given. At 3 days of life, the patient was given a second dose of pulmonary surfactant due to the persistent respiratory distress. Oxygen demand hereby decreased with FiO_2_ of 0.5. Chest radiograph showed diffuse decreased transmittance with air bronchograms in the right and left lower lung lobes (Figure 1C). At 4–6 days of life, clinical condition was relatively stable with mild reduction of FiO_2_ from 0.45 to 0.40. Meropenem was discontinued at 6 days of life.

**Figure 2:**
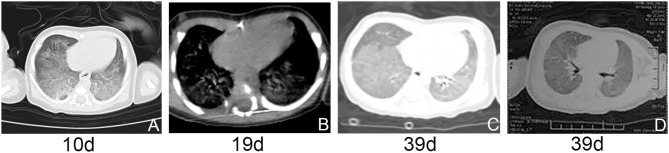
**(A–D)** HRCT and airway reconstruction showed decreased transmittance and ground glass opacity in bilateral lungs.

At 7 days of life, due to the improved conditions, the patient was extubated and instead given NCPAP with FiO_2_ of 0.35. At 10 days of life, a high-resolution computed tomography (HRCT) scan revealed diffuse ground glass opacity in bilateral lungs (Figure 2A). At 13 days of life, he was intermittently given NCPAPC with FiO_2_ of 0.21. During 14–17 days of life, he was supplied with humidified high-flow oxygen (3 L/min) via nasal cannula. At 18 days of life, dyspnea with hypoxemia reoccurred and NCPAP was hereby reinitiated with FiO_2_ of 0.25. On 15–19 days of life, dexamethasone was given at a dose of 0.3 mg/kg/day but no improvement was observed. HRCT scan revealed decreased transmittance and ground glass opacity in most of lung lobes (Figure 2B). Several laboratory test results were anormal, including neutrophilic leukocytosis (leukocytes 18.630/mm^3^; neutrophils 11.790/mm^3^), mildly elevated C-reactive protein concentration (28.6 mg/L), and procalcitonin concentration (3.6 mg/L). Cefoperazone-sulbactam sodium was administered, showing no effect.

Given the recurrent hypoxemic respiratory failure and ground glass opacity in both lungs, we highly suspected ILD. With the informed consent from the parents, 2-ml blood samples were collected from the neonate and the parents for genetic testing.

**Figure 3:**
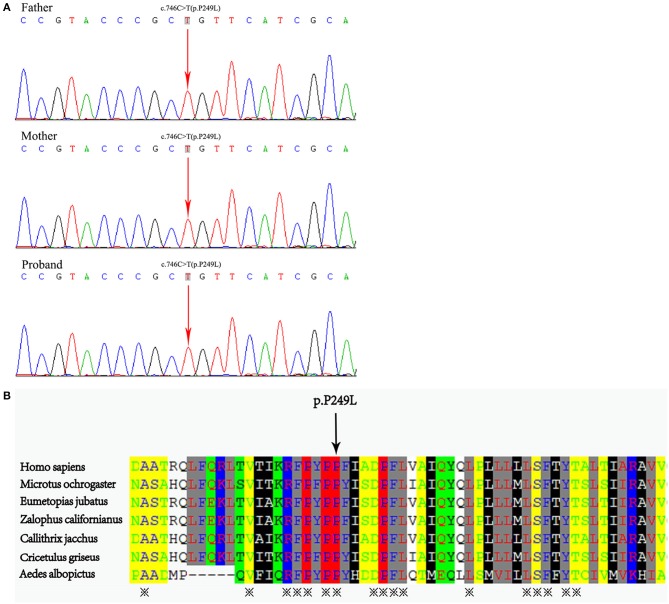
**(A)** Whole exome sequencing demonstrated a homozygous missense mutation C-to-T transition at position 746 in exon 8 of the *ABCA3* gene in the neonate, which resulted in a Pro249Leu mutation. The mutations were detected in his parental DNA, respectively. **(B)** Protein alignment showed conservation of the P249 residue of *ABCA3* across seven species.

The genetic analysis revealed a homozygous missense mutation (c.746C>T, p. P249L) in the coding region of exon 8 of *ABCA3* gene in the neonate. Sanger sequencing verified that both parents were heterozygous carriers of this mutation (Figure 3A). The mutation occurred in an evolutionarily conserved site by cross-species comparison of *ABCA3* protein sequences (Figure 3B).

At 20 days of age, the treatment strategy was adjusted as follows: intravenous methylprednisolone pulse therapy at a dose of 10 mg/kg/day (3-days courses on a monthly basis), together with azithromycin at a dose of 10 mg/kg every other day, and hydroxychloroquine at a dose of 5 mg/kg/day. At 30 days, chest radiograph showed patchy increased density shadows in bilateral lungs (Figure 1D). At 39 days of age, airway reconstruction and HRCT scan revealed decreased transmittance, ground glass opacity, and mildly reduced lesion extent (Figures 2C,D). During the aforementioned medications, the patient consistently depended on supplemental oxygen.

**Figure 4:**
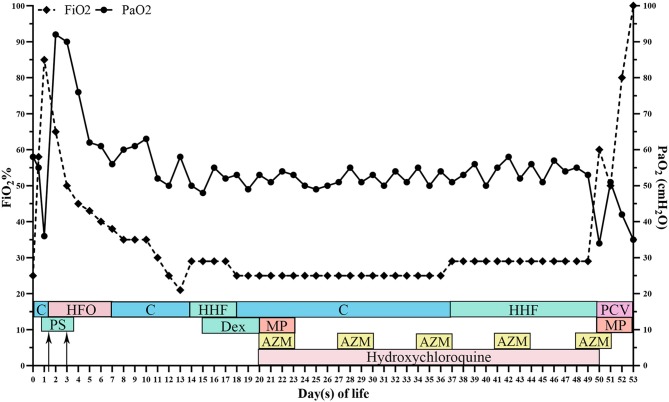
The overall time curve of medication, respiratory support, arterial oxygen partial pressure, and fraction of inspire oxygen. FiO_2_, fraction of inspire oxygen; PaO_2_, arterial oxygen partial pressure; C, continuous positive pressure ventilation; HFO, high-frequency oscillatory ventilation; HHF, humidified high-flow oxygen; PCV, pressure controlled ventilation; Dex, dexamethasone; MP, methylprednisolone; AZM, azithromycin; PS, pulmonary surfactant.

At 50 days of life, the patient was transferred to a lung transplantation center. Due to the extremely severe hypoxemia with PaO_2_ of 24 mmHg, mechanical ventilation with FiO_2_ of 0.6 was given. Chest radiograph showed diffuse decreased transmittance, air bronchogram, and vesicular shadows in the right and left lower lung lobes (Figure 1E). At 52 days of life, he had frequent episodes of cyanosis with oxygen saturation decreased to <50%, and meanwhile, the FiO_2_ gradually elevated to 0.8. At 53 days of life, further rescue was stopped due to the parental declination and the patient was discharged from the hospital. The autopsy request was declined by the parents as well. The main treatment course is demonstrated in Figure 4.

## Discussion

The *ABCA3* gene maps to chromosome 16p13.3 and is highly expressed in the limiting membrane of lamellar bodies of type II alveolar epithelial cells. ABCA3 transports the phospholipids into lamellar bodies, where these lipids, together with the surfactant proteins, assemble into pulmonary surfactants. The newly synthesized surfactants are then released into the alveoli. The *ABCA3* mutation could influence a set of physiological processes (7, 8): (1) phospholipid transportation, (2) lamellar body formation, (3) SP-B and SP-C transcription and translation, (4) pulmonary surfactant assembly and structural transformation in type II alveolar epithelial cells, (5) pulmonary surfactant production, and (6) lung epithelial cell apoptosis.

Numerous studies have indicated that *ABCA3* mutation is one of the major causes of neonatal fatal RDS and ILD in children (9, 10). *ABCA3* mutation is inherited as an autosomal recessive. More than 200 cases of *ABCA3* mutations have been reported, most of which are compound heterozygotes (11). Kröner et al. conducted a retrospective study on 1,153 patients with ILD. Of these patients, 242 were sequenced for *ABCA3* gene, and at least one variation was identified to be shared in 69 patients. In addition, two pathogenic mutations were identified in 40 patients (12). Shulenin et al. reported that 16 in 21 infants with RDS at birth carried *ABCA3* gene mutations (13). El Boustany et al. reported a premature newborn with severe RDS in a few hours after birth, and sequencing analysis revealed two frame shift mutations p.pro1301argfs ^*^45 and p.eu1695argfs^*^103 in *ABCA3* gene (14). López Castillo et al. reported a full-term female newborn with RDS immediately after birth, and the homozygous nonsense mutation c. 4681C>T in *ABCA3* gene was detected (15).

A classification scheme of childhood interstitial lung disease (chILD) was elaborated by the chILD Research Network (16). ILD in neonate has a high early mortality. Due to ILD's non-specific clinical manifestations and its progressive deterioration and early lethality, earlier identification of ILD in neonate is extremely difficult but meaningful.

Diagnostic tests for chILD include echocardiography, imaging studies (chest X-ray, thin-section CT) pulmonary function test, bronchoscopy with BAL, genetic test, and lung biopsy. Considering many ILD cases at infant and children stages have genetic pathogenesis, genetic test is hereby recommended for rapid diagnosis. According to the guidelines formulated by the American Thoracic Society, gene diagnosis was listed as one of the main diagnostic tests in infant with ILD, and it is recommended to be performed prior to lung biopsy. In particular, cases with clinical and radiological changes are suspected with inborn problems in surfactant metabolism, which can also be confirmed by genetic tests. If the sequencing results are negative, lung biopsy with electron microscope will be performed (17). In this case, bronchoscopy with BAL was recommended, but it was not performed due to clinical instability and rapid disease progression. In addition, lung biopsy was canceled due to the parental declination. Despite the aggressive treatment, the patient was still unable to wean from supplemental oxygen. The characteristic radiological findings made us highly suspect ILD and led to the immediate decision of genetic testing.

In this study, a homozygous mutation (c.746C>T) in *ABCA3* gene was detected in the neonate's genome. Family sequencing analysis concluded that the two mutant alleles were inherited respectively from the phenotypically healthy parents. Pathogenicity analysis was conducted according to the “interpretation standards and guidelines for gene sequence variation” formulated by ACMG and AMP in 2015 (6): (1) The mutation (c.746C>T, Pro249Leu) is located in a mutation hotspot (PM1) where mutations such as Asp253His, Asp253Tyr, Pro248Leu, and Pro248Ser have been reported. (2) The frequency of this variation in the normal population database is 0.00010, a low-frequency variation (PM2). (3) SIFT, Mutation Taster, GERP++, REVEL all predicted that this mutation was harmful. (4) The neonate's clinical manifestations and radiological changes were highly consistent with the phenotype of ILD caused by *ABCA3* gene mutations. Therefore, the evidence intensity of c.746C>T was “PM1+PM2+PP3+PP4,” which was judged as a possible pathogenic variation. Having reviewed the HGMD, 1,000 Genomes and EXAC gene databases, we confirmed that the mutation is a novel *ABCA3* homozygous mutation.

The neonate's clinical phenotypes, imaging findings, and genetic results strongly support the diagnosis of ILD caused by *ABCA3* mutation. The homozygosity of the mutation may explain the severity of clinical status. However, given the absence of lung histopathology, the pathogenicity of the mutations remains unelucidated.

There are no standardized treatment guidelines for chILD. In current practice, methylprednisolone, hydroxychloroquine, and azithromycin were widely used, though their pharmacological mechanisms and clinical effects remain inconclusive. In the study conducted by Kroner et al., of all the 35 *ABCA3*-mutated patients treated with systemic steroids, eight displayed moderate improvement, and two received complete remission. On the other hand, the administration of hydroxychloroquine resulted in good responses in three cases, and moderate improvement in six cases, of all the 18 patients (14). El Boustany et al. reported that a premature newborn carrying *ABCA3* mutation was given high doses of methylprednisolone intravenously plus oral administration of hydroxychloroquine and azithromycin but received no improvement (17). A similar futile case was also reported by López Castillo et al. on a full-term newborn carrying *ABCA3* mutation (15). So far, only isolated or a small number of cases have been reported, with varying prognosis. Based on the current inadequate data, we think that systemic steroid could be a double-edged sword, due to the fact that details such as recommended dosages and doses remain unclear. Hence, clinical trials with larger number of such patients are urgently needed to establish more effective therapeutic intervention guidelines. Currently, lung transplantation, an option for end-stage lung disease, may be the only fundamental solution for ILD. However, the rapid development of gene editing technologies may cast light on ILD treatment in the future.

## Data Availability Statement

All datasets generated for this study are included in the article/supplementary material.

## Ethics Statement

This study was carried out in accordance with the Ethical Review Committee of the Zibo Central Hospital. Written and informed parental consent was obtained for publication of this case report.

## Author Contributions

MW designed the study, collected data, drafted the initial manuscript, and carried out the initial analyses. LM reviewed and revised the manuscript. HF and AH collected and collated clinical data of the patient.

## Conflict of Interest

The authors declare that the research was conducted in the absence of any commercial or financial relationships that could be construed as a potential conflict of interest.

## Abbreviations

ABCA3: ATP-binding cassette transporter subfamily A member 3
NRDS: neonatal respiratory distress syndrome
PS: pulmonary surfactant
ILD: interstitial lung disease
FiO_2_: fraction of inspired oxygen
PaO_2_: arterial oxygen partial pressure
HRCT: high-resolution computed tomography
chILD: childhood interstitial lung disease.
